# Healthcare Professionals’ Role in Social Media Public Health Campaigns: Analysis of Spanish Pro Vaccination Campaign on Twitter

**DOI:** 10.3390/healthcare9060662

**Published:** 2021-06-02

**Authors:** Ivan Herrera-Peco, Beatriz Jiménez-Gómez, Juan José Peña Deudero, Elvira Benitez De Gracia, Carlos Ruiz-Núñez

**Affiliations:** 1 Nursing Department, Faculty of Medicine, Alfonso X El Sabio University, Villanueva de la Cañada, 28691 Madrid, Spain; bgomejim@uax.es; 2 Faculty of Health Sciences, Alfonso X el Sabio University, Villanueva de la Cañada, 28691 Madrid, Spain; jpenadeu@uax.es (J.J.P.D.); eben@uax.es (E.B.D.G.); 3 High Resolution Hospital, APES Poniente, Av. Tierno Galván, Loja, 18300 Granada, Spain; cruiznu@gmail.com

**Keywords:** COVID-19, healthcare professionals, public health, vaccines, social media

## Abstract

The COVID-19 pandemic has generated a great impact worldwide both on the population health but also on an economic and social level. In this health emergency, a key element has been and still is the need for information, which has become a daily concern for many people. Social media represent powerful tools for searching and gathering health-related information, thus becoming a new place where health authorities need to be present to disseminate information of preventive measures like vaccines against COVID-19, as well as try to block information against these public health measures. The main goal of this study was to analyze the role that healthcare professionals have in Twitter to support the campaign of public institutions on vaccination against COVID-19. To address this study, an analysis of the messages sent on Twitter containing the hashtag #yomevacuno, between 12 December 2020 was developed using the NodeXL software (Social Media Research Foundation, Redwood, CA, USA), focusing on content analysis of tweets and users’ accounts to identify healthcare professionals. The results show that healthcare professionals represent only 11.38% of users, being responsible for 6.35% of impressions generated by the network #yomevacuno. We can observe that traffic information generated by healthcare professionals is not significant in comparison with institutions (*p* = 0.633), but it is compared to common users (*p* = 0.0014). The most active healthcare professionals were pharmacists (40.17%), nurses (27.17%), and physicians (12.14%). Their activity (90.43% of messages) was mainly focused on sharing messages generated by other users’ accounts. From original content generated by healthcare professionals, only 78.95% had a favorable storytelling on the vaccine, but without sharing information about vaccines or vaccination. As a conclusion for this study, the participation of healthcare professionals in the dissemination and generation of information within the #yomevacuno communication strategy, led by the Spanish Ministry of Health, has been scarce. We emphasize the need to enhance communication skills in social networks to support public health campaigns through these increasingly important social media.

## 1. Introduction

COVID-19 disease, which started in Wuhan, China, with the first case reported on December 2019 and continuing today, was defined by World Health Organization (WHO) as an international outbreak of a public health emergency and declared as a pandemic on 11 March 2020 [1]. This disease has a number of characteristics that have facilitated its rapid spread, such as the long incubation period [2] and the high number of asymptomatic carriers [3]. The impact of this pandemic worldwide, regarding the level of deaths and infected individuals is indisputable [4], but one of the aspects that should also be taken into account is the impact on mental health caused not only by the disease, but also by all the measures implemented to curb and/or prevent its transmission, including the generation of information and its dissemination [5,6].

As has been described in other health emergencies, individuals may generate negative emotions resulting in stress, anxiety, fear, or uncertainty [7,8], not to mention irritability, anger, frustration, phobia, fear, and intolerance [9,10].

One of the elements that affects individuals’ mental health and, in addition, may lead to a decrease in adherence to recommendations by health authorities to address the struggle against COVID-19, is the information consumed by the population [10,11]. Importantly, in health emergencies such as that caused by the COVID-19 pandemic, the need for information has become a daily concern for many people [12].

The term ‘infodemic’ is a mixture of “information” and “epidemic” and refers to an abundance of information which can be either accurate or inaccurate. According to the World Health Organization (WHO), COVID-19-related ‘infodemic’ is as dangerous as the virus itself. One of the fastest ways to obtain health-related information is from the internet and, in particular, within social media [13]; not to mention the information spread through messaging apps such as WhatsApp, Telegram, etc. [14], elements that are essential to understand the problem of current ‘infodemic’. Social media represent powerful tools for searching for health-related information or for gathering such information. It is in this aspect in which it can evidently be seen that the population in general, and especially patients, share information, or opinions, about subjects related to health [15].

An example of the importance of this type of media for the spread of information is the social network Twitter, which has approximately 271 million users being responsible for over 500 million tweets every day [16]. While other social networks, such as Facebook, Instagram, Tiktok, etc., do provide health information, Twitter is the one playing the most important role in disseminating information during the COVID-19 pandemic [17,18,19,20].

However, both the easy and quick access to these platforms and the lack of control over the veracity of the content posted, mean that they can be considered as rapid means of dispersing unverified health information [20,21], constituting a potential threat to public health [12,21] since disinformation, misinformation, and conspiracy theories hinder mitigation, transmit misleading messages about the disease, and promote ineffective precautionary measures [22,23].

‘Infodemic’ cannot be eliminated, yet it can be managed [24]. The rapid detection of health misinformation is essential for such management and it involves appropriate training in evidence-based practice, together with a good strategy for dissemination of information [25], which helps the population to be well informed and able to effectively react to a pandemic [26].

In an emergency situation where public health is at risk, the role of healthcare professionals as key elements for communication strategies, based upon reliable and verified information, proves essential [27,28]. In this sense, healthcare professionals would become active agents in spreading information and controlling false information, either misinformation or disinformation, in order to protect the public from wrong contents. Thus, increasing empowerment and health promotion, and playing a crucial role in supporting individuals and communities into the understanding of public healthcare messages [29,30].

The main objective of this work is to analyze the role of healthcare professionals during the start of the campaign launched in the social network Twitter, by the Ministry of Health of the Government of Spain, in favor of vaccination against COVID-19. More specifically, what type of professionals participated, their role in the impact instilled by the campaign, and the type of messages they conveyed through this social network.

## 2. Materials and Methods

### 2.1. Study Design and Ethics

An observational, retrospective, time-limited study was proposed, in which activity on the social network Twitter was analyzed.

This study was considered exempt from ethical review because it was performed upon a social network and the study did not interfere with any patient or human data beyond measuring internet activity among Twitter users. Also considering that this study only compiled data from users who consented on Twitter to disclose their data openly (i.e., no privacy settings were selected by users) being completely public.

Furthermore, users’ accounts have been anonymized in order to develop good research practices in social networks [31].

### 2.2. Data Collection

The information from the tweets was extracted through an API (application programming interface) search tool, using the professional version of the software NodeXL (Social Media Research Foundation, Redwood, CA, USA).

To achieve the objectives proposed in this study, the keyword “yomevacuno” (‘igetvaccinated’) and the hashtag #yomevacuno were selected. The main reason for this selection was that this is the very hashtag used by Spanish health authorities to start a support campaign for anti-COVID-19 vaccines and the vaccination itself, as the best way to stop the spread of COVID-19 and raise awareness on the utility of vaccines. Although COVID-19 is a pandemic, we believe that it is necessary to analyze the situation across countries, in a specific manner, because the social situation in each country has its own particularities. For this reason, this study focuses on an observational analysis of the network ‘yomevacuno’ in Spain, excluding users that could communicate in Spanish on Twitter but are located in a different place from Spain.

The Twitter users included in the analysis of the data were those who had sent tweets with the above-mentioned characteristics during a predefined period. Unverified users were also included, to analyze the dissemination of messages.

The tweet selection criteria for this study were: (i) tweets published in Spanish language; (ii) tweets containing the hashtag #yomevacuno or the keyword “yomevacuno” or the phrase “yo me vacuno”; (iii) users located in Spain; (iv) tweets that were published between 14 December (00:00 a.m. CET) and 28 December 2020 (23:59 p.m. CET).

With the data collected from the hashtag #yomevacuno, it was observed that a total of 3038 Twitter users participated, amounting for 915,736 impressions (visualization and interaction with tweets). In addition, it was found that there were a total of 4918 interactions, including 421 (8.56%) tweets (considered as original content), 2377 retweets (48.33%), 126 replies (2.56%), and 1994 mentions (40.54%)

### 2.3. Data Analysis

The analysis of the data obtained was performed in several steps. The first step was to analyze the most influential Twitter users who posted under the aforementioned hashtag, as well as their characteristics. We have used a traditional social network analysis technique like the betweenness centrality score (BCS). This centrality measure in social network terms, is associated with the user’s power in the network, understanding it like the importance of connecting and transmitting information across the entire network [32]. The BCS measures the influence of a vertex over the flow of information to other vertices, always assuming that information will travel through the shortest vertex path. The BSC value reflects how a user can control the information, choosing whether to share it or not, disclosing it to his/her network [33,34]. In our study, the BCS is the value used to define the influential users in the network #yonomevacuno. The Twitter users are compiled and grouped by nodes using the Clauset–Newman–Moore cluster algorithm.

In relation to the hypothetical activity in the network ‘yomevacuno’, the BCS allows us to identify the content, activities, and/or influential users that would be strongly associated with overall Twitter activity measured by the metrics of total tweets, impressions, retweets, and replies [35]. It is important to define that tweets are associated with the creation of original content by another user, meanwhile, retweets are an indicator that shows the transmission of a tweet sent by another user (it is not original content). Finally, the impression is an indicator of information propagation obtained when the number of tweets is multiplied by the number of followers [35].

Finally, an analysis of both the users’ account description and the contents of the tweets was performed. With regard to users’ accounts, we analyzed the description of users identifying as healthcare professionals (HCP from now). Furthermore, original tweets analysis was taken into account, since these are deemed to be the ones generating the original content disseminated throughout the user network. Prior to the content analysis of original tweets, the coding variables were defined. The first variable, ‘media’, captured the presence of media in the tweet and the type of media employed (i.e., video, image, or document), if applicable. The variable ‘message function’ was coded using three coding variables: ‘information’, ‘action’, or ‘community’; where ‘information’ means tweets which main purpose was to inform, educate, or update the reader on COVID-19 transmission, symptoms, or how vaccines work. ‘Action’ tweets were intended to prompt changes in the behavior of other Twitter users. ‘Community’ tweets were associated with stories from members of the community about community-building, vaccines, or COVID-19 disease experience.

Finally, content credibility of tweets was performed, where researchers analyzed the existence of external links that allowed independent corroboration, and analysis of the structure of tweets’ searching for clues about possible failures in credibility like inappropriate wording, spelling, and/or grammar [20].

The content coding was performed independently by two researchers and corroborated by a third person, so that any approach and focus differences were always discussed and resolved with full agreement.

### 2.4. Statistical Analysis

This study is quantitative and observational. For data statistical analysis, descriptive and inferential statistics, we used the Statistical Package for the Social Sciences software (SPSS) version 23.0. (IBM, Armonk, NY, USA) Kolmogorov–Smirnoff non-parametric analysis was performed for comparison of means. The statistical level of significance was set at *p* < 0.05.

## 3. Results

### 3.1. Users Analysis

**Figure 1:**
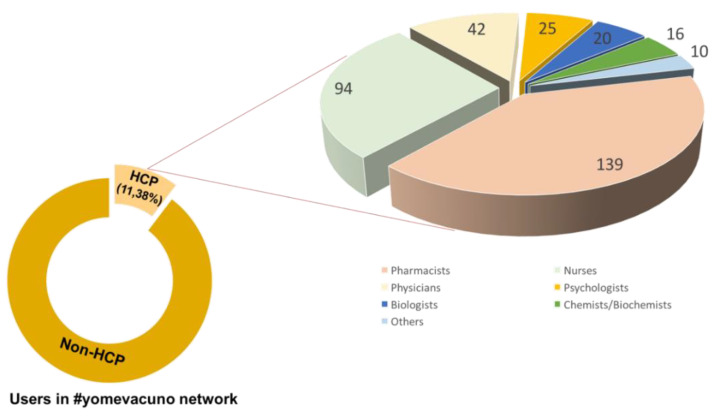
Healthcare professionals in ‘yomevacuno’ network. Where HCP, means healthcare professionals, and non-HCP means users than do not define themselves as healthcare professionals (including public and private institutions and organizations).

Within the #yomevacuno network, 3038 users were found, of which 346 (11.38%) identified themselves as healthcare professionals in their user description. Within these users, identified as healthcare professionals, it was observed that the four professional groups with the highest activity were pharmacists, 139 users (40.17%); nurses, 94 users (27.17%); physicians, 42 users (12.14%); and psychologists, 25 users (7.23%) (see Figure 1).

In relation to the messages sent through the ‘yomevacuno’ network by healthcare professionals, 397 messages were generated, 38 of which were tweets (9.58%), 181 retweets (45.59%), 10 replies (2.52%), and 168 mentions (42.31%) (Table 1), with a creation of original content.

The hashtag #yomevacuno was analyzed and the users that participated in this network were ranked by the betweenness centrality score, finding that the 10 most influential users were mainly accounts of official organizations, six out of the 10. The remaining user accounts were individual profiles, three of which belonging to healthcare professionals (Table 2).

### 3.2. Influence of Healthcare Professionals in Potential Impressions in ‘Yomevacuno’ Network

With regards to the influence of users labeled as healthcare professionals within the hashtag studied, #yomevacuno, it is found that they generated an amount of 58,177 impressions on the network, which represented 6.35% of the total impressions (Table 3). While the rest of users in this network were identified as non-healthcare professional and generated 93.65% of total impressions in the network ‘yomevacuno’. From this group, users identified as institutions generate 54.45% of impressions with an average of 356.2 interactions per user (Table 3). We observed that the impressions from HCPs compared with non-HCPs is not significant (*p* = 0.129), likewise when comparing HCPs with institutions (*p* = 0.99), meanwhile the impressions generated by HCPs against common users present a significant difference (*p* = 0.0014).

Within the healthcare professionals, it was observed that those generating the highest number of impressions were pharmacists with 22,808 (39.2%), followed by physicians 14,819 (25.47%), nurses 11,041 (18.98%), psychologists 3332 (5.73%), and others (biologists, biochemists, etc.) with 2490 impressions (10.62%).

### 3.3. Content Analysis

We proceeded to analyze all the tweets generated by healthcare professionals on the twitter network under the hashtag #yomevacuno, finding that 78.95% (30) had a favorable storytelling on the vaccine, 15.79% (six) did not generate an opinion nor provide information as they only sent the hashtag #yomevacuno, and 5.26% (two) were tweets not associated with healthcare information of any kind. From 30 original tweets we observed that 12 (40%) were associated with the category ‘inform’, 11 (36.7%) were messages included in ‘actions’, and seven (23.7%) were messages defined as ‘community’.

**Figure 2:**
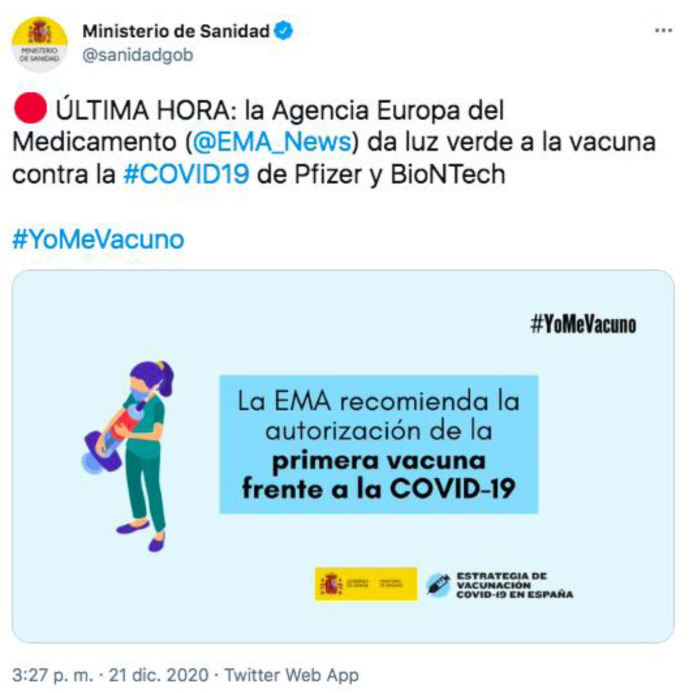
Image of the most shared tweet by healthcare professionals in #yomevacuno network.

The data show us that the main activity of healthcare professionals in the network ‘yomevacuno’ was associated with retweets (Table 1), 181 messages that represent 45.6% of total messages from healthcare professionals. It was noted that the most widespread messages were related to the announcement by the Spanish Ministry of Health on the approval of the Pfizer vaccine by the EMA (repeated 63 times) (Figure 2), and the statement by the official account of the Spanish Government on the start of vaccination in Spain (repeated 44 times). With regards to the provision of truthful information and reliable sources about the safety of vaccines, the message came from the Spanish Vaccinology Association and was repeated on 11 occasions (Table 4).

In relation to the analysis of the credibility perceived in healthcare professionals’ tweets, we found that only three tweets (5.26%) offered external links that allowed independent corroboration; in addition, we observed presentation problems (inappropriate wording, spelling, and/or grammar) in 27 tweets (71.05%).

## 4. Discussion

In the present study, the role of HCPs in the dissemination and generation of content on Twitter was evaluated by analyzing #yomevacuno, focused on boosting the vaccination awareness campaign against COVID-19 in Spain.

It is important to highlight that the participation within social media, and Twitter particularly, have a voluntary nature. This situation means that HCPs participate at different level adopting specific roles based in their experience in social media. We could have a mixture of lurkers, observers, passive users, and of course, active contributors. Non-participant users [36] continue to belong to the network ‘yomevacuno’ and they have potential access to important information related to vaccines and pro-vaccine news. Situation that could explain the low participation of HCPs in traffic information in the network analyzed.

As can be seen, individual users, HCPs and non-HCPs, had a lower weight than institutions within all the traffic, impression generated in the analyzed network, something that contrasts with analyses performed in other vaccination campaigns, such as the one carried out in 2018 for influenza vaccination in Spain [22] or international campaigns on awareness of public health issues, where individual users were the main generators of information and traffic in the networks versus institutions [26,33]. However, it is remarkable that HCPs have a more important role in traffic generated in the network ‘yomevacuno’ than common users, since our findings according to the previous bibliography, suggest that health authorities should appeal to HCPs’ social responsibility to attract them as followers for the messages (tweets) that can be generated in public health campaigns, so that the messages can have more dissemination and more credibility, as well as the campaign itself [37].

The common role of HCPs in social media is focused on activities of a personal nature rather than professional nature [30,35]. This can be linked with the low participation of users identified as healthcare professionals in the dissemination of information, that can be considered as a work activity [30] for vaccination awareness, and it could be explaining why HCPs have more weight in traffic information than common users in the network ‘yomevacuno’.

In relation with HCP’s messages in the network ‘yomevacuno’, there exist a low number of tweets (original content). Situation that could be associated with the low level of followers that could be observed in HCPs Twitter accounts, being a common strategy trying to retweet health messages from other sources, like public health institutions, rather than tweeting themselves [36].

Likewise, it was observed that the messages generated by healthcare professionals did not provide relevant information on either vaccines or the situation at that point regarding COVID-19. This situation contrasts with other studies in which it was observed that healthcare professionals used the network to send reassuring messages, or to provide understandable information to the public on specific measures and situations related to the COVID-19 pandemic [38,39].

Although there are numerous messages on the network in favor of the vaccination campaign, it can be noted that a high percentage of them have a political and communicative nature, with a low number of messages with scientific content and providing information on the vaccines themselves and their usefulness. When analyzing the tweets, as messages with original content, it can be found they do not meet many of the elements that Zubiaga et al. [20] defined as important elements that a tweet must have to be considered reliable. That is, tweets, in order to be considered reliable information, must present the characteristics of authority, support, independent corroboration, and presentation (appropriate writing, spelling, and grammar) [20], and text plausibility [20,40,41]. After analyzing the data collected, it was found that the original tweets from healthcare professionals did not offer independent corroboration and even the presentation failed, triggering poor credibility perceptions by users.

This situation, coupled with the increased stress and anxiety levels described during the pandemic [17,42] and the users’ lack of trust towards information coming up from official institutions in times of great social confusion [19] as occurs in the COVID-19 pandemic [7,8,43], raise the need for reference figures, not associated with national or international healthcare institutions.

It is important to point out that healthcare professionals are considered by the population as an essential element for the understanding of health-related messages [29] and their absence in social networks as reference elements may generate distrust and even disaffection towards truthful healthcare information [42], not preventing the spread of antivaccine messages [19]. This situation can make it easy for network users to be redirected to irrelevant information about the importance of vaccination or, worse, to be redirected to inaccurate or false information about vaccines in general, and vaccination against COVID-19 in particular [18].

This study has some limitations. Firstly, the social network Twitter has been assessed, which limits the analysis of the campaign to its users. Secondly, we analyzed messages sent in Spanish from users geolocated in Spain and, moreover, the categorization of healthcare professionals was based on themselves presenting as such, which means the number of healthcare professionals may be underestimated, as there are many who do not wish to state their profession in their profiles.

However, we want to highlight an important strength of this study, because to the knowledge of the authors, the present study is the first to address an analysis of the role of healthcare professionals how key elements of a pro-vaccination campaign, against the COVID-19, in the Spanish speaking community in Twitter. This study is the first and will be able to allow initiate further developments focused to improve the efficacy of future public health campaigns.

## 5. Conclusions

It is of paramount importance that healthcare professionals understand the need for being present on social networks from a professional point of view, so that they can become central elements in the dissemination and creation of reliable information from a scientific point of view, aimed at health care.

Although it is extremely important for public institutions to be present and lead these campaigns, we believe it is very important to bear in mind that these institutions should not prompt rejection in a certain part of the population, and this is where healthcare professionals can perform as reference figures to which users of social networks can turn to, in order to obtain healthcare information.

Another important element observed in this study is the low generation of original content and, in addition, the generated tweets had the problem of not providing links to external sources, with 71% presenting issues of inappropriate writing, spelling, and/or grammar, making an impression of low reliability.

We believe that this situation suggests the need to implement training actions for healthcare professionals on the use of social networks to enhance their participation and improve the effectiveness of communication. It is important to focus these actions on showing how to prepare reliable tweets, focusing on the plausibility of content, attaching external and reliable sources, and taking care of tweets writing.

We consider it necessary that healthcare professionals, as individual users, can actively collaborate in the dissemination of public health prevention policies through social networks.

## Figures and Tables

**Table 1:** Messages from healthcare professionals in #yomevacuno network.

	‘Yomevacuno’ Network	Healthcare Professionals	%
Tweets	421	38	9.03
Retweets	2377	181	7.61
Replies	126	10	7.94
Mentions	1994	168	8.42

Note: %, is percentage.

**Table 2:** Top ten influential users ranked in #yomevacuno network by their betweenness centrality score (which measure their influence over the flow of information in the network).

Rank	Account Description	Betweenness Centrality Score
Pos1	Official account of Spanish Ministry of Health	2,374,284.987
Pos2	Official account of European Medicine	944,199.663
Pos3	Official account of Spanish Agency of Medicines and Medical Devices	917,876.405
Pos4	Official account of Spanish Government	553,721.661
Pos5	Healthcare professional (pharmacist)	433,595.884
Pos6	Healthcare professional (virologist)	177,106.144
Pos7	Healthcare professional (physician)	160,118.699
Pos8	Official account of European Commission	152,567.392
Pos9	Citizen (journalist)	82,384.904
Pos10	Citizen (politic)	77,850.270

**Table 3:** Impressions generated by users identified in #yomevacuno network.

Impressions			Test		
Total	%	Average; SE	Z; *p*-Value		
58,177	6.35	109.02; 11.05	0.871; 0.129 (n.s.)		
857,559	93.65	189.54; 3.65			
Institutions	498,655	54.45	356.2; 6.08	0.367; 0.633 (n.s)	
Users	358,904	39.2	31.16; 1.97		0.999; 0.001 ***

Note: HCP means healthcare professionals. S.E. means standard error. % means percentage. N.s. means no significance. *** means statistical significance (*p* < 0.001).

**Table 4:** Most shared tweets by healthcare professionals in #yomevacuno network.

Rank	Times Shared (%)	Message	Type of Media	Message Function	Original Source
1	63 (17.55%)	*ÚLTIMA HORA: la Agencia Europa del Medicamento (@EMA_News) da luz verde a la vacuna contra la #COVID19 de Pfizer y BioNTech #YoMeVacuno* -BREAKING NEWS: the European Medicines Agency (@EMA_News) gives the green light to #COVID19 de Pfizer y BioNTech #YoMeVacuno- (Figure 2)	Image	Update	Spanish Minister of Health
2	44 (12.25%)	*La vacunación en España comenzará el 27 de diciembre, el primer día hábil acordado con los socios europeos para iniciar este proceso. La vacuna será suministrada de manera gratuita en la red sanitaria habitual. Lo explica el ministro de @sanidadgob,* Salvador Illa. #YoMeVacuno Vaccination in Spain will begin on December 27th, the first working day agreed with European partners to start this process. The vaccine will be provided free of charge through the usual health network. It is explained by the Minister of @sanidadgob, Salvador Illa. #YoMeVacuno	Official video statement of the Minister of Health	Update	Spanish government official account
3	20 (5.57%)	*La vacunación en España comenzará el domingo 27 de diciembre” @salvadorilla. #YoMeVacuno #VacunaCOVID19* Vaccination in Spain will begin on Sunday, December 27” @salvadorilla. #IVaccinateMe #VaccineCOVID19	Video	Update	Spanish Minister of Health
4	11 (3.06%)	*Desde la @AEV_Vacunas han creado un DECÁLOGO para hablar sobre la necesidad de la vacuna contra #COVID #YoMeVacuno* From the @AEV_Vaccines have created a DECALOGUE to talk about the need for the vaccine against #COVID #YoMeVacuno	Document	Information	Spanish Association of Vaccinology
5	11 (3.06%)	*Por qué las #vacunas son seguras. Porque cuentan con las garantías de vigilancia a gran escala.Estudios y ensayos de seguimiento Informes frecuentes de seguridad. Máxima transparencia #YoMeVacuno @EU_Commission* Why #vaccines are safe. Because they have the guarantees of large-scale surveillance. Follow-up studies and trials Frequent safety reporting.Maximum transparency #YoMeVacuno @EU_Commission	Document	Information	Spanish Agency of Medicines and Medical Devices

## Data Availability

Data available on request from the corresponding author.
